# Multiscale characterization of microstructural reconfiguration induced by oxidation in lignite from the Lingquan mining colliery

**DOI:** 10.1038/s41598-025-03996-4

**Published:** 2025-07-02

**Authors:** Jian Chen, Ying Wen, Baoshan Jia, Xiaopeng Mao, Qinai Zhou

**Affiliations:** 1 School of Mining, Liaoning Technical University, Fuxin Liaoning, 123000 China; 2 School of Safety Science and Engineering, Liaoning Technical University, Fuxin, 123000 Liaoning China

**Keywords:** Lignite, Low-temperature oxidation, Pore structure, Functional group, Microcrystalline structure, Coal, Energy

## Abstract

The microstructure changes of Lingquan mine oxidized lignite at different oxidation temperatures and the influence of low-temperature oxidation on the spontaneous combustion characteristics of lignite is studied in this paper. The pore structure characteristics, surface morphology, microcrystalline structure, and functional groups of lignite in Lingquan Mine were studied using low-temperature nitrogen adsorption, SEM, XRD, and FTIR tests. The research results indicate that characteristics of mesoporous pore structure and the peak areas of functional groups of oxidized lignite are significantly different from those of original lignite. The mesoporous volume of oxidized lignite is 183.65–306.61% times that of original lignite. The mesoporous specific surface area of oxidized lignite is 140.11–180.56% times that of original lignite. The peak area of the -CH_3_ functional group of oxidized lignite in the Lingquan mine is 122.68–252.74% times that of the original lignite. The peak area of the C = O functional group of oxidized lignite is 120.09–239.78% times that of the original lignite. The aromatic layer spacing *d*_002_ of oxidized lignite increased by 0.20–1.12% compared to the original lignite. Compared with the original lignite, the average size La of the aromatic layer of oxidized lignite increased by 4.10–11.45%. The quantity of aromatic layers *M*_*c*_ of oxidized lignite decreased by 2.31–12.37% compared with the original lignite. The stacking height *L*_*c*_ of the aromatic layer of oxidized lignite decreased by 2.13–11.39% compared with the original lignite. The coalification degree *P* of oxidized lignite P decreased by 1.72–9.85% compared with the original lignite. This research revealed that the mesoporous volume and specific surface area of oxidized lignite in Lingquan Mine increased significantly, the surface became rougher, and the oxidation reaction space and contact area increased. The peak area of oxidized lignite -CH_3_ and C = O functional groups improved significantly, enhancing the reaction activity. The spacing and average size of aromatic layers of oxidized lignite are more significant than those of original lignite. However, there are fewer aromatic layers, resulting in a looser microcrystalline structure, lower stability of molecular structure, and degree of coalification. In summary, oxidized lignite in Lingquan Mine is more prone to spontaneous combustion than original coal.

## Introduction

**Fig. 1:**
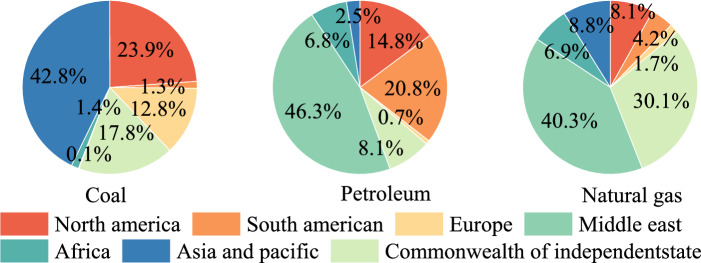
Global proved reserves of coal, oil, and natural gas.

Coal is essential in meeting global energy consumption as a significant fossil fuel^1–3^. Global coal demand is expected to rise due to rising natural gas prices and economic recovery following the COVID-19 pandemic^4^. Fossil fuels are exceedingly unevenly distributed around the world. According to statistics from the 2022 edition of the"BP Global Energy Statistical Yearbook"^5^, as shown in Fig. 1. The proven coal resource reserves are 143,197 million tons, accounting for 13.3% of the world’s total, and natural gas reserves are 8.4 trillion cubic meters, accounting for only 4.5% of global natural gas reserves in China, as seen in Fig. 1. The distribution characteristics of fossil fuels in China are characterized by"more coal, less oil, and less gas."Coal consumption will account for 56% of the total primary energy consumption in 2021, indicating that coal is still the dominant energy source in China^6,7^. China’s proven lignite reserves, as per the third national coal survey, total 130 billion tons (13% of total coal reserves), while the energy landscape remains stable in the short term^8^. In particular, 77.1% of lignite is distributed in northeastern Inner Mongolia, China’s most extensive lignite base^9^. Layered mining in thick coal seams (> 10 m) leads to repeated oxidation of residual coal in upper goafs during lower-layer extraction, altering physicochemical structures and intensifying spontaneous combustion through enhanced oxidation activity^10–12^.. Spontaneous coal combustion poses severe threats to mining safety, ecological security, and economic stability while resulting in irreversible waste of non-renewable resources^13–16^..

Many researchers have conducted relevant experiments and studies in the past decade to determine the changing characteristics of spontaneous combustion in coal after it is oxidized at different temperatures. Wang et al.^17^ studied the effect of pre-oxidation on the spontaneous combustion behavior of coal under different oxygen concentrations at a fixed temperature of 80 °C. They found that the low-temperature oxygen consumption of oxidation coal increased with the help of thermogravimetry, infrared spectroscopy, and scanning electron microscopy. Wang et al.^18^ confirmed that the surface structure of oxidation coal is looser, the spontaneous combustion characteristics are enhanced, and the oxygen-containing groups in the molecular structure of coal are increased. Shi et al.^19^ used XPS, TG-DSC, and SEM experiments to study the effect of the pre-oxidation process on the spontaneous combustion characteristics of Shendong low-order long-flame coal. It is concluded that when the pre-oxidation temperature reaches and exceeds the critical temperature of 200 °C, the specific surface area and total pore volume of the coal sample begin to decrease significantly. At the same time, the specific surface area of the coal sample treated at 70 °C is more significant than that of the raw coal, and the heat released increases, which increases the risk of coal spontaneous combustion. Lu et al.^20^ used a new test platform to study the thermal runaway characteristics of lignite in Ordos. The results show that the increase in oxidation level causes the hardness of coal to decrease and is conducive to the collapse of the structure, increasing the probability of spontaneous combustion of oxidation coal. Zhang et al.^21^ used TG-FTIR infrared spectroscopy combined experiments to study the primary oxidation process of oxidized coal and raw coal. The experimental results prove that the reaction activation energy of oxidized coal is lower than that of raw coal, and the risk of spontaneous combustion of oxidized coal is higher. Ma et al.^22^ used STA-FTIR and in-situ FTIR methods to study oxidation coal. The content of -CH_3_ increased during the pre-oxidation process, and the risk of spontaneous combustion of pre-oxidized coal increased. It is worth noting that oxidized coal at 120 °C has the highest risk of spontaneous combustion during secondary oxidation^23–25^.

In addition, to analyze the thermal behavior of oxidation coal, Lü et al.^26^ used TG and DSC experiments to investigate the oxidation reaction of coal. It is found that the oxidation reaction of oxidation coal occurs earlier than that of raw coal, the combustion rate and short-term combustion intensity are also higher than that of raw coal, and there is a critical value of oxidation temperature. When the oxidation temperature is lower than the critical value, oxidation coal’s risk is higher than raw coal’s. At the same time, the research results of Tang et al.^27^ revealed that after pre-oxidation treatment, the spontaneous combustion tendency of oxidation coal is higher than that of raw coal, especially in the early stage of low-temperature oxidation. Although the content of aliphatic hydrocarbon groups in oxidized coal is small, it is compensated by the increased content of oxygen-containing functional groups. This is one of the reasons why oxidized coal has a strong tendency to ignite spontaneously. In addition, Liu et al.^28^ studied the differences between the spontaneous combustion characteristics of bituminous coal and raw coal in the 30–180 °C range when the pre-oxidation temperature was 90 °C and 140 °C. In the slow oxidation stage, raw coal’s oxygen consumption and heat release intensity are lower than oxidized coal’s, and the risk of spontaneous combustion of oxidized coal is higher. However, many researchers also found that the oxidation activity of coal decreased after pre-oxidation^29–33^.

In other words, the pre-oxidation temperature is essential to coal’s oxidation reactivity and spontaneous combustion. When the pre-oxidation temperature is lower than the critical temperature, pre-oxidation treatment will increase the risk of coal spontaneous combustion. However, when the pre-oxidation temperature exceeds this critical temperature, pre-oxidation treatment can reduce the functional group content and the risk of spontaneous coal combustion^16^. The self-ignition properties of coal are affected by various factors, including physical and chemical structure^34–36^. The pore structure and gas flow characteristics of coal may be affected when coal is subjected to high-temperature oxidation^37,38^. It can also alter chemical groups, affecting the reaction between coal and oxygen^39–41^.

Existing studies have predominantly examined coal’s characteristic temperatures, thermal behaviors, and chemical changes during pre-oxidation, while neglecting microstructural evolution (surface morphology, microcrystalline structures, active functional groups) and temperature-dependent spontaneous combustion mechanisms in Lingquan Mine’s oxidized lignite, leaving critical theoretical gaps for effective fire prevention.

The research aims to elucidate the oxidation-induced alterations in lignite’s microstructural characteristics and their mechanistic implications for spontaneous combustion propensity. Therefore, Cretaceous low-rank lignite was selected as the experimental sample and pre-oxidized at 50, 100, 150 and 200℃ in this study. The physical structure of raw and oxidized coal samples, including pore characteristics and surface morphology, was studied using low-temperature nitrogen adsorption and SEM. In addition, the chemical structure of these coal samples, including surface element composition, functional groups, and microcrystalline structure, was studied through FTIR and XRD analysis. The research results provide a theoretical basis for determining the spontaneous combustion characteristics of pre-oxidized coal, thereby guiding the formulation of coal mine fire prevention measures.

## Experimental materials and testing methods

### Preparation of coal sample

This paper selects lignite from the Cretaceous coal seam in eastern Mongolia as the research object. Fresh coal samples were collected following the national standard"Methods for Determination of Physical and Mechanical Properties of Coal and Rocks"(GB/T23561.1–2009), sealed, and sent to the laboratory. The oxide layer on the coal surface was peeled off, and only the central part was selected before the experiment. Coal samples are crushed and screened in an air atmosphere to obtain pulverized coal with a particle size of 0.075–2.00 mm, which is sealed and stored in a brown bottle to avoid light according to the national standard"Preparation Method of Coal Samples"(GB474-2008). Pulverized coal was oxidized in an air atmosphere using a programmed temperature control system to obtain pre-oxidized coal samples.

**Fig. 2:**
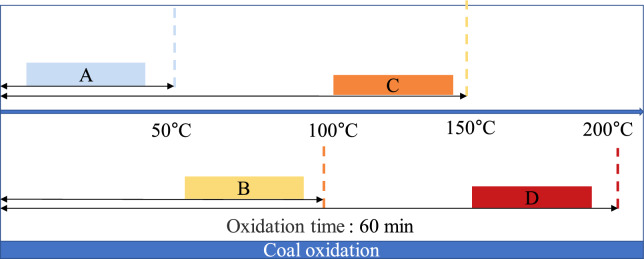
Oxidation process of coal samples at different oxidation temperatures.

The fresh coal samples were oxidized at 50, 100, 150, and 200 °C for 60 min, respectively, when the dry air flow rate in the oxidation furnace was 100 ml∙min^−1^. The coal sample is naturally cooled to room temperature in the air environment, and then the coal sample heating device is turned off. The oxidation process of lignite in Lingquan Mine is shown in Fig. 2. The numbers of raw lignite and oxidized lignite from Lingquan Mine are shown in Table 1. The industrial analysis results of oxidized lignite at different oxidation temperatures are shown in Table 1.

**Table 1:** Industrial analysis of oxidized lignite.

Serial number	Oxidation temperature (℃)	Industrial analysis (%)				Tendency of spontaneous combustion
		M_ad_	A_ad_	V_ad_	FC_ad_	
RC	0	3.59	35.96	27.26	33.19	II
A	50	3.44	36.31	26.56	33.69	
B	100	2.35	36.81	26.39	34.45	
C	150	2.65	37.67	24.60	35.08	
D	200	1.97	41.68	20.02	36.33	

Note: M_ad_—a mass fraction of the moisture of the air-dry base, %; A_ad_—a mass fraction of the ash of the air-dry base, %; V_ad_—a mass fraction of the volatile fraction of the air-dry base, %; FC_ad_—a mass fraction of the fixed carbon of the air-dry base, %.

### FTIR and TG testing

The infrared spectrum of pre-oxidized coal was tested by a Japanese Shimadzu IRPrestige-21 Fourier transform infrared spectrometer. The change rules of surface functional groups of coal samples before and after oxidation were obtained through numerical calculation. Instrument working environment and working parameters: power supply voltage is 75–265 V, and frequency is 45–67 Hz. The ambient temperature is 18–35℃. The KBr tableting method needs to be used to prepare the sample, and the coal sample of test and KBr are mixed at a mass ratio of 1:150 before sample testing. The mixed sample was ensured to be thoroughly mixed in an agate mortar. It is pressed into a translucent uniform sheet under the pressure of 20MPa. The wave number range of the instrument scan is 400–4000 cm^−1^, and the number of scans is 45 times.

Thermogravimetric analysis (TGA): TGA measurements were performed on a NETZSCH STA 449 F3 thermal analyzer. The coal sample (1–3 mg) was heated from ambient temperature 28 °C to 400 °C under a flowing air atmosphere 60 mL/min at a constant heating rate of 20 °C/min.

### SEM and low-temperature nitrogen adsorption testing

The morphological characteristics of the surface of pre-oxidized coal sample particles were explored through a JSM-7500 F field emission scanning electron microscope in high vacuum mode. The pore parameters, including pore volume, specific surface area, average pore diameter, and pore size distribution characteristics, were tested, and a fully automatic specific surface area and pore size analyzer based on the adsorption method was adopted. This experiment aims to explore the evolution of coal micropore structure characteristics during the pre-oxidation process. It is concluded that the low-temperature nitrogen adsorption method is more accurate in characterizing mesopores 2–50 nm based on relevant literature^42^ and repeated experiments simultaneously. This paper uses high-purity liquid nitrogen as the adsorbent to conduct nitrogen adsorption and desorption measurements within the liquid nitrogen temperature of 77 K and the relative pressure of 10^–6^−1. In addition, the BET and BJH^43^ methods were used to determine the specific surface area and pore size distribution characteristics, respectively. The BET^44^ equation is shown in Eq. (1).

1 $$P/[V(P_{0} - P)] = 1/(V_{m} \cdot C) + [(C - 1)/V_{m} \cdot C](P/P_{0} )$$

In Eq. (1), *P* represents adsorbate partial pressure; *P*_0_ represents saturated vapor pressure; *V* represents absorption amount; *V*_m_ represents the volume of a single layer; *C* represents a dimensionless constant.

In addition, the BJH equation is shown in Eqs. (2)-(4)^45^:

2 $$r_{k} = - 4.14(\log P/P_{0} )^{ - 1}$$

3 $$t = - 4.30[5/\ln P/P_{0} ]$$

4 $$r = r_{k} + t$$

In Eq. (4), *r*_*k*_ represents Kelvin radius; *t* represents the thickness of the adsorption layer; *r* represents the pore radius.

### XRD testing

The XRD-6100 X-ray diffractometer was used to conduct XRD characterization of pre-oxidized coal samples. XRD test parameters: the receiving target is Cu-Kα, the scanning angle is 10–75°, and the scanning speed is 5°∙min^−1^. The working voltage is 40kV, and the current is 40 mA. Data were analyzed by Jade software. The spectral peak positions and characteristics of the aromatic crystallites of oxidized lignite in Lingquan Mine are similar to those of graphite crystals. Therefore, the microcrystalline structure of oxidized lignite from Lingquan Mine was analyzed by comparing the XRD patterns of oxidized lignite in Lingquan Mine and graphite. The XRD spectrum of oxidized lignite can be fitted into three peaks: γ peak, 002 peak, and 100 peak, which respectively reflect side chains of aliphatic hydrocarbon, stacking height of aromatic lamella *L*_c_, and the average size of layers of aromatic lamella *L*_a_ according to relevant literature^46–49^. In addition, coal P is used to describe the accumulation structure of the aromatic and fatty layers^47^. The parameters of the microcrystalline structure of oxidized lignite from Lingquan Mine were calculated according to Eqs. (5)-(10)^50–52^.

5 $$d_{002} = \frac{{\uplambda }}{{{\text{2sin}}\theta_{{{002}}} }}$$

6 $$d_{100} = \frac{{\uplambda }}{{{\text{2sin}}\theta_{{{100}}} }}$$

7 $$L_{c} = \frac{0.94\lambda }{{\beta_{002} \cos \theta_{002} }}$$

8 $$L_{a} = \frac{1.84\lambda }{{\beta_{100} \cos \theta_{100} }}$$

9 $$M_{c} = \frac{{L_{{\text{c}}} }}{{d_{{{002}}} }}$$

10 $$P = \frac{{{3}{\text{.975 - }}d_{{{002}}} }}{{{3}{\text{.975 - 3}}{.354}}} \times 100\%$$

In formulas (5)-(10), λ represents the X-ray wavelength of Cu-Kα, and the λ value is 1.5405 Å; *β*_002_ and *β*_100_ represent the half-maximum width of the 002 peak and the 100 peak, respectively, rad; *θ*_002_ and *θ*_100_ represent the diffraction angles corresponding to the 002 peak and the 100 peak respectively, °. *d*_002_ represents the aromatic layer spacing, Å; *L*_c_ represents the stacking height of aromatic lamella, Å; *L*_a_ represents the average size of layers of aromatic lamella, Å; *M*_c_ represents the number of aromatic lamella stacked; *P* represents the degree of coalification, %.

## Results and discussion

### Nitrogen adsorption curves and microscopic morphology

**Fig. 3:**
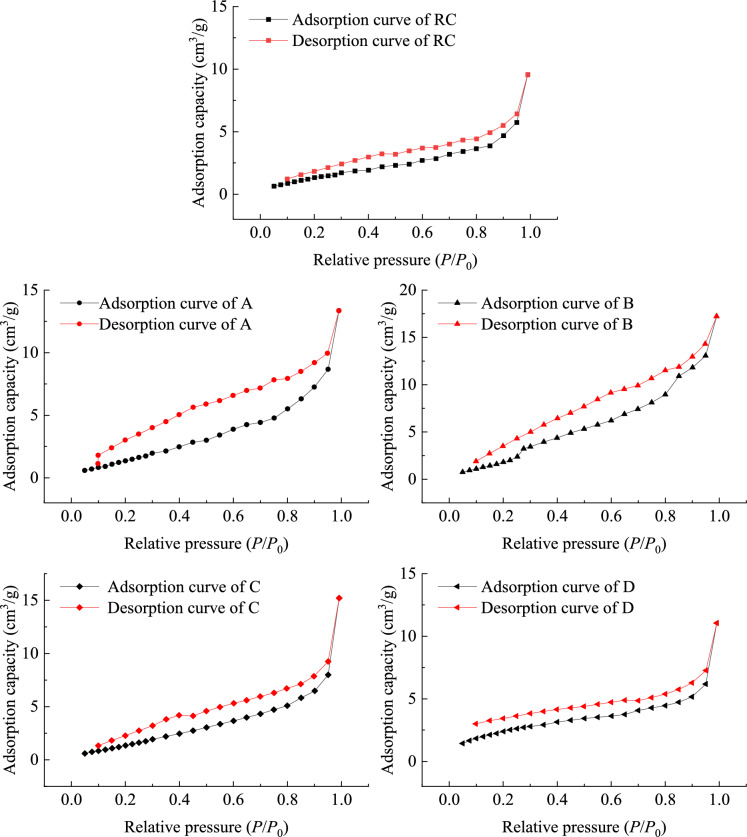
Nitrogen adsorption–desorption curve of isotherm of oxidized lignite at low temperatures in Lingquan Mine.

Nitrogen molecules undergo single-layer adsorption, multi-molecular layer adsorption, and capillary condensation in the pores in sequence. These phenomena occur in the opposite order during the low-temperature nitrogen adsorption and desorption processes^53^. The low-temperature nitrogen adsorption and desorption characteristics of oxidized lignite in Lingquan Mine were systematically studied according to the adsorption and desorption curves of RC, A, B, C, and D coal samples, as shown in Fig. 3. It can be seen from Fig. 3 that the amount of nitrogen adsorption increases with the increase of nitrogen partial pressure (*P*/*P*_0_). The nitrogen adsorption of oxidized lignite increases relatively slowly, with single-layer adsorption being the main form and the adsorption rate being relatively small when the relative pressure is 0.1 < *P*/*P*_0_ < 0.5. The pores of oxidized lignite are dominated by multi-layer adsorption when the relative pressure is 0.5 < *P*/*P*_0_ < 0.8, and the capillary condensation phenomenon begins to appear. The amount of nitrogen adsorption increases compared with the previous period. Nitrogen adsorption is mainly capillary condensation when relative pressure 0.8 < *P*/*P*_0_ < 1.0 and the amount of nitrogen adsorbed begins to increase significantly. Meanwhile, the nitrogen adsorption isotherm curve also becomes steeper. In other words, the rate of nitrogen adsorption is faster, and the capacity of nitrogen adsorption is more substantial.

**Fig. 4:**
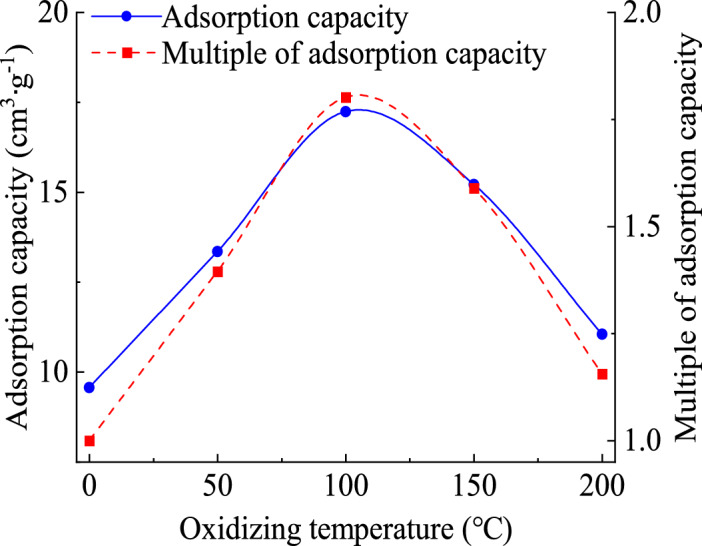
Changes curve of nitrogen adsorption amount of oxidized lignite at low temperatures in Lingquan Mine.

It can be concluded that the maximum amount of nitrogen adsorption of raw lignite is 9.57 cm^3^·g^−1^, and the maximum amount of nitrogen adsorption of oxidized lignite A, B, C, and D are 13.35, 17.24, 15.21 and 11.05 cm^3^·g^−1^ respectively, as shown in Fig. 4. The maximum nitrogen adsorption capacity of oxidized lignite shows a trend of first increasing and then decreasing as the oxidation temperature increases. The maximum adsorption of oxidized lignite is 1.80 times that of the original coal, and the minimum is 1.16 times that of the original coal. The research results show that the adsorption space of oxidized lignite in Lingquan Mine is larger, and the adsorption capacity is stronger. The fundamental reason for the change in the amount of nitrogen adsorption is the evolution of the oxidized lignite pore structure, and detailed analysis results are as follows. The water in lignite evaporates and precipitates as the oxidation temperature increases. The number of mesopores increases, the volume of total pores increases, and the maximum amount of nitrogen adsorption increases. Then, the mesopores and macropores shrink and collapse as the oxidation temperature rises. The total pore volume decreases, and the maximum nitrogen adsorption decreases.

In summary, the adsorption–desorption isotherms of oxidized lignite in Lingquan Mine all have hysteresis loops^54^. Based on hysteresis loop classification (IUPAC guidelines), ​both raw coal and oxidized lignite exhibit Type H3 hysteresis loops. The research results indicate that the wider the hysteresis loop, the better the pore connectivity^55^. The width of the hysteresis loop of oxidized lignite shows an evolution from wide to narrow, the complexity of the pore type shows a changing trend from simple to complex, and the strength of pore connectivity shows a changing pattern from strong to weak as the oxidation temperature increases^56^. The pore of oxidized lignite has better connectivity, greater capacity for nitrogen adsorption, looser structure, and greater risk of coal spontaneous combustion compared with raw coal, as shown in Fig. 3 and Fig. 4.

**Fig. 5:**
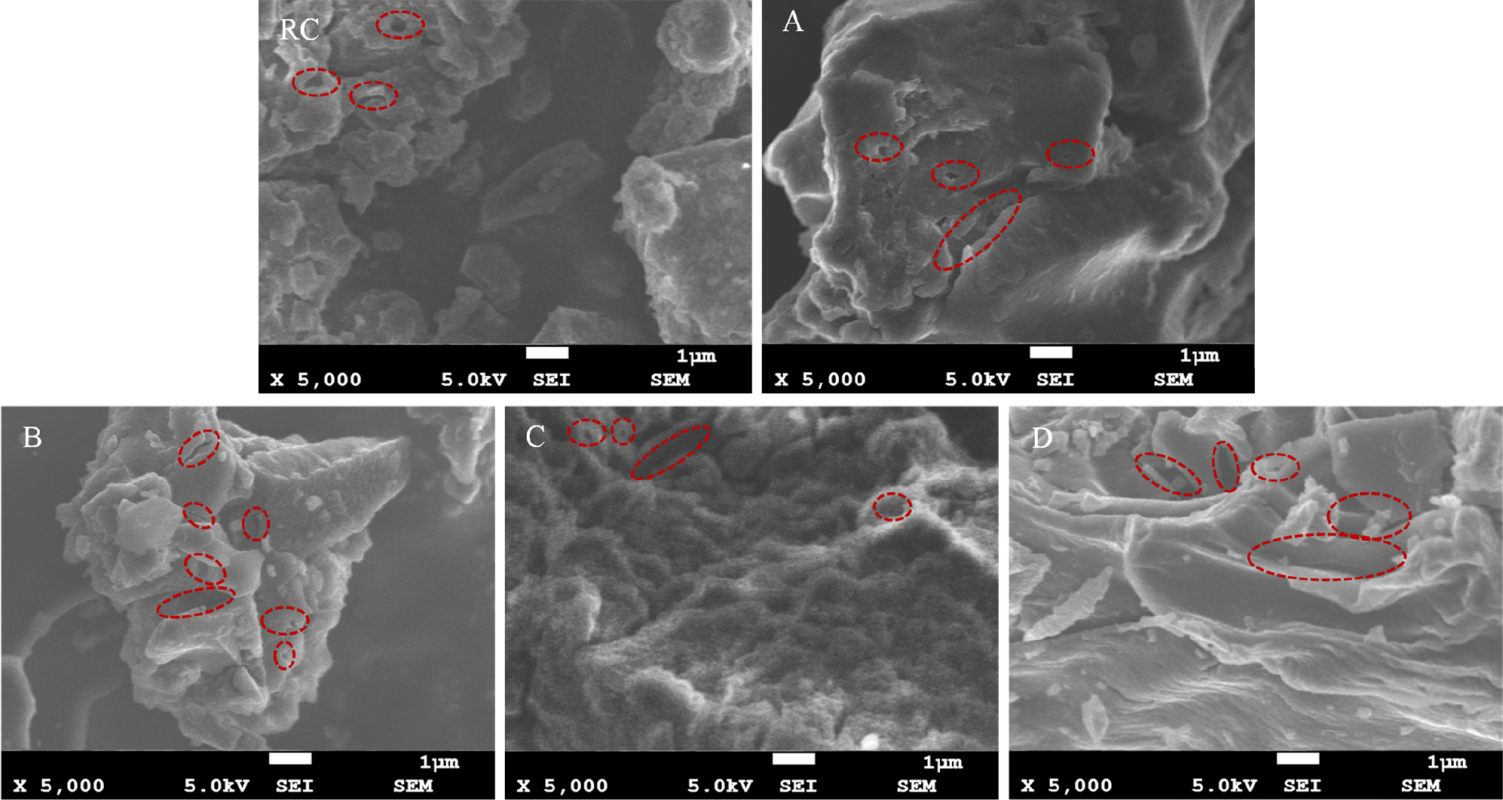
Microscopic morphology of oxidized lignite in Lingquan Mine.

The research found that the vast majority of pores in the oxidized lignite of Lingquan Mine are irregular pores through SEM tests. The macropores and cracks in oxidized lignite are marked according to the SEM image scale, as shown in Fig. 5. Notably, the number of pores oxidized lignite A and B is more significant than that of raw coal. The pore structure of oxidized lignite C collapsed, and the number of pores decreased. The shrinkage of the pore structure intensifies as the oxidation temperature continues to increase, and the number of pores in oxidized lignite D continues to decrease. The oxidized lignite in Lingquan Mine undergoes thermal stress and expansion, so the oxidized lignite’s pore structure becomes looser and porous. The penetration and adsorption of oxygen in oxidized lignite becomes more muscular, and heat accumulates more easily. Therefore, the risk of spontaneous combustion of oxidized lignite in Lingquan Mine is greater than that of raw coal.

### Evolution characteristics of pore structure

#### Distribution characteristics of mesoporous pores

**Fig. 6:**
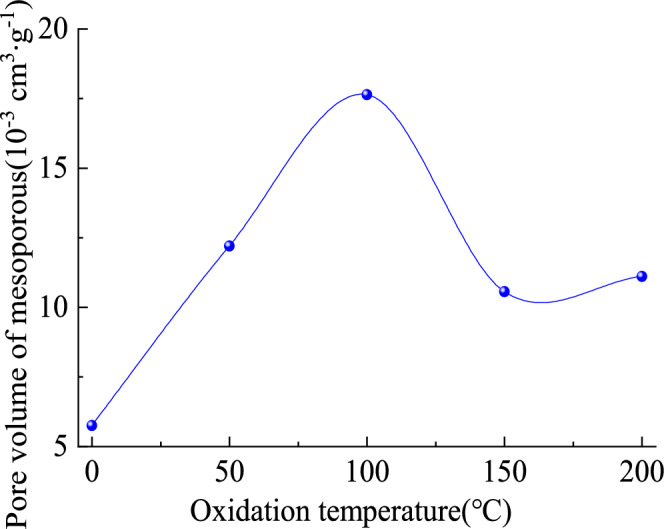
Variational curve of the mesoporous volume of lignite with increasing oxidation temperature.

The mesoporous volume of oxidized lignite in Lingquan Mine shows a nonlinear evolution trend of first increasing, then decreasing, and then increasing again, as shown in Fig. 6. Detailed research results are as follows. When the lignite oxidation temperature is 0–100 °C, the water evaporates and escapes, and with the increase of oxidation temperature, larger pores appear. The thermal stress on the mesoporous structure is minor, and the pore structure remains relatively intact without collapse. The mesoporous volume of oxidized lignite reaches a maximum value when the oxidation temperature is 100 °C. The oxidation temperature continues to increase, and the stress on the mesopores increases when the oxidation temperature range is 100–200 °C. The mesoporous volume distribution range of oxidized lignite is 0.01056–0.01763 cm^3^∙g^−1^. The mesoporous pore volume of coal sample B in oxidized lignite is 3.07 times that of raw coal. The mesoporous pore volume of coal sample C in oxidized lignite is the smallest, 1.84 times that of raw coal. In summary, the mesopores of the oxidized lignite from Lingquan Mine are more developed, which can provide more reaction space for oxygen than the original coal sample, and the risk of spontaneous combustion is greater. In addition, the evolutionary trends of mesopore structure of oxidized lignite are consistent with the evolution trend of mesoporous volume. The accuracy and reliability of the research results were verified.

#### Distribution characteristics of specific surface area of mesopore

**Fig. 7:**
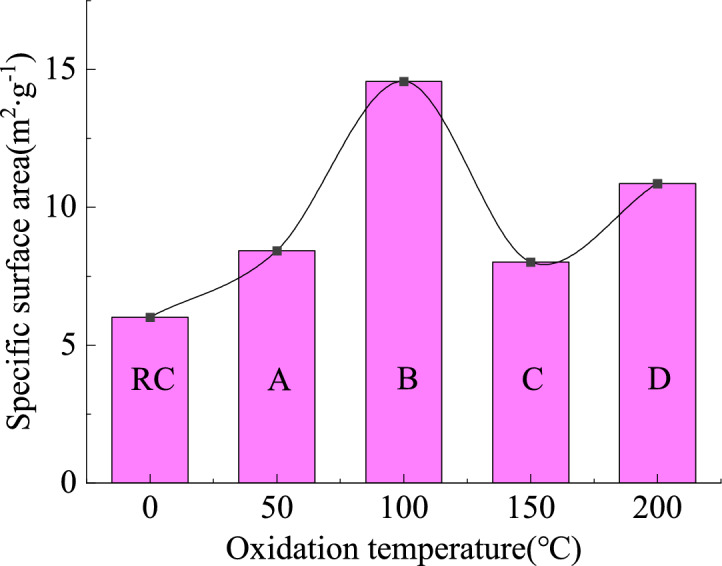
Variational curves of mesoporous specific surface area of oxidized lignite.

The specific surface area of the mesopore of oxidized lignite in Lingquan Mine shows a nonlinear evolution pattern of first increasing, then decreasing, and then increasing, which is consistent with the change rule of its mesoporous volume, as shown in Fig. 7. This trend is consistent with the evolution law of pore connectivity of oxidized lignite that first becomes stronger and then weaker^20^. In addition, oxidized lignite’s pore-specific surface area range is 8.02–14.57 m^2^·g^−1^. Oxidized lignite B has the largest specific surface area of mesopore, which is 2.42 times that of raw lignite. Oxidized lignite C has the smallest specific surface area of mesopore, 1.33 times that of raw lignite.

This research reveals that low-temperature oxidation promotes the complex evolution of the pore structure of lignite in Lingquan Mine, increasing the specific surface area of the mesopore of oxidized lignite and increasing the risk of spontaneous combustion of lignite^57^.

**Fig. 8:**
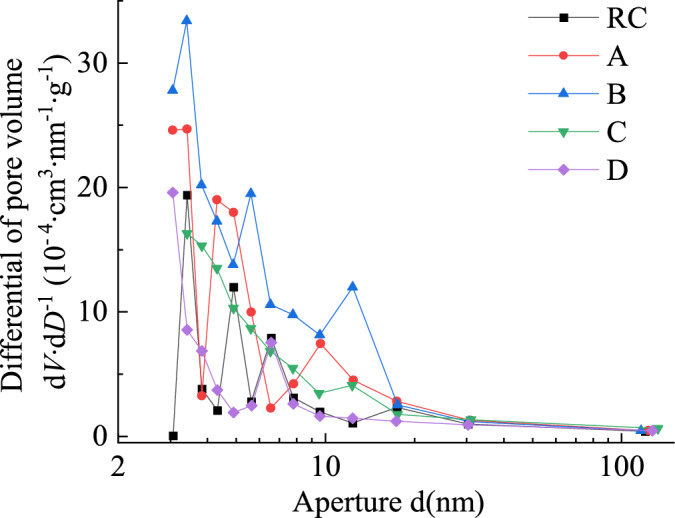
Differential distribution of pore volume variation between raw coal and oxidized lignite.

Figure 8 shows the differential variation curves of pore volume differential versus pore size for raw coal and oxidized lignite. Compared to raw coal, oxidized lignite exhibits significant differences in the pore size distribution peaks within the 2–50 nm range. The distribution peaks of oxidized lignite in this range are generally higher than those of raw coal, a trend consistent with the increases in specific surface area and pore volume of oxidized lignite as oxidation temperature rises. Such microstructural evolution is posited to elevate the propensity for spontaneous combustion in goaf-hosted coal, likely attributable to enhanced oxygen adsorption capacity and accelerated exothermic reactions within the expanded mesoporous network.

#### Distribution characteristics of fractal dimension of mesopore

The Frenkel-Halsey Hill (FHH) model was used to research the isotherm curves of low-temperature nitrogen adsorption and desorption of oxidized lignite in Lingquan Mine and obtain its surface and spatial fractal dimensions. Domestic and foreign scholars commonly use the FHH model, which has been proven to be the most effective, reliable, and scientific model^58–60^, as shown in the Eq. (11).

11 $$\ln \left( {\frac{V}{{V_{0} }}} \right) = C + A\left[ {\ln (\ln \frac{{P_{0} }}{P})} \right]$$

In formula (11), *V* represents the amount of adsorption of gas molecules at equilibrium pressure *P*, ml·g^−1^; *V*_0_ represents the amount of adsorption of the monolayer, ml·g^−1^; *P* represents pressure of adsorption equilibrium, MPa; *P*_0_ represents the saturated vapor pressure of adsorption, MPa; *C* represents dimensionless constant; *A* represents the factor of fractal dimension.

Draw the relationship curve between ln(*V/V*_0_) and ln(ln(*P*_0_*/P*)), and perform linear fitting on the curve. The fractal dimension D is calculated based on the slope A and its relationship with the fractal dimension.

The adsorption behavior is dominated by van der Waals force when *P*/*P*_*0*_ is less than 0.5, and the equation is as shown in ((12).

12 $$A = (D - 3)/3$$

The adsorption behavior is dominated by capillary force when *P*/*P*_0_ is greater than 0.5, and the calculation formula of fractal dimension is shown in Eq. (13).

13 $$A = D - 3$$

However, the D value calculated by Eq. (12) is usually less than 2 in actual solutions, which violates the geometric meaning of the pore fractal dimension. The value of D calculated from Eq. (13) is in the range of 2–3^61^. This paper uses Eq. (13) to calculate the fractal dimension, considering the simultaneous force of significant capillary condensation inside the pores. Detailed research results are shown below.

The FHH curve can be divided into two stages, and the dividing point is *P*_0_*/P* = 0.5. The surface fractal dimension D_FFD_ is obtained through numerical calculation when the relative pressure *P*_0_*/P* is less than 0.5. D_FFD_ represents the roughness of the pore surface. The larger the D_FFD_ value, the rougher the pore surface of the oxidized lignite in Lingquan Mine. The spatial fractal dimension D_SFD_ is calculated when the relative pressure *P/P*_0_ is greater than 0.5. D_SFD_ represents the complexity of the pore structure. The larger the D_SFD_, the more complex the pore structure of the oxidized lignite in Lingquan Mine is^62^.

**Fig. 9:**
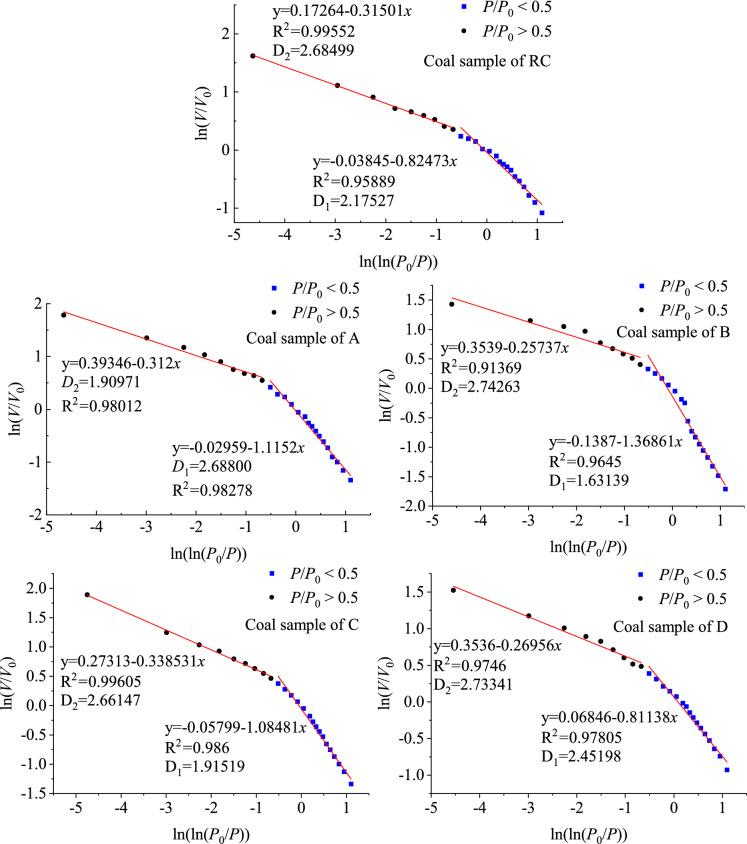
Fractal dimension of oxidized lignite in Lingquan Mine.

**Fig. 10:**
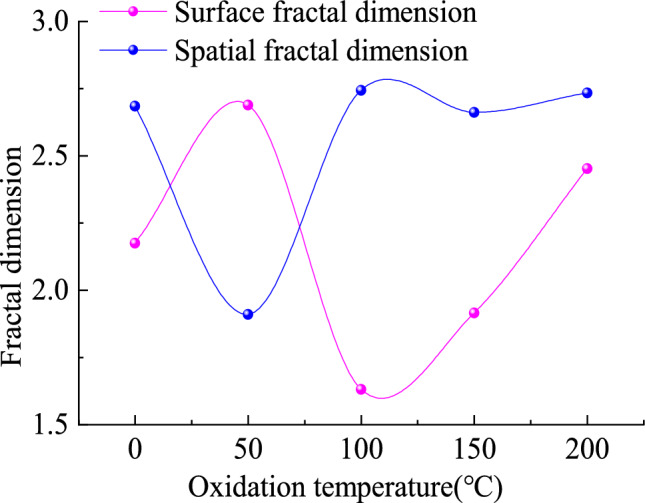
Variational curve of surface fractal dimension of oxidized lignite in Lingquan Mine.

The surface fractal dimensions of oxidized lignite A, B, C, D, and raw coal in Lingquan Mine are 2.68800, 1.63139, 1.91519, 2.45198, and 2.17527 respectively, as shown in Fig. 9. The surface fractal dimension of the oxidized lignite in Lingquan Mine shows a trend of first decreasing and then increasing, which is opposite to the overall variation trend of mesoporous volume and specific surface area of mesopore, as shown in Fig. 10. The surface fractal dimensions of oxidized lignite A and D increased by 19.07% and 11.29%, respectively. Compared with the original coal, the surface fractal dimensions of B and C decreased by 33.34% and 13.58%, respectively. The research results show that the pore surface of oxidized lignite becomes smoother after low-temperature oxidation treatment at 100 and 150 °C, and the pore surface doesn’t have significant fractal characteristics. The pore surface of oxidized lignite becomes rougher after low-temperature oxidation treatment at 50 and 200 °C, and the surface fractal shape becomes more significant.

The spatial fractal dimension of the oxidized lignite in Lingquan Mine shows a trend of first increasing, then decreasing, and then increasing, consistent with the variational trend of mesoporous volume and specific surface area of mesopore, as shown in Fig. 10. The spatial fractal dimensions of oxidized lignite B, C, and D increased by 20.69%, 18.27%, and 20.42%, respectively, while the spatial fractal dimensions of A decreased by 13.91%, compared with the original coal. The research results show that the pore space structure of oxidized lignite becomes more complex after low-temperature oxidation treatment at 100, 150, and 200 °C, and the pore structure has significant spatial fractal characteristics. The pore structure of oxidized lignite becomes simpler after low-temperature oxidation treatment at 50 °C and does not have significant spatial fractal characteristics.

Mechanistic Evolution of Fractal Dimensions During Low-Temperature Oxidation. The temperature-dependent evolution of surface and spatial fractal dimensions reveals three distinct regimes. During Stage I (0–50 °C), incipient oxidation and pore activation dominate. Limited oxidative modification preserves the native pore architecture, retaining intrinsic surface roughness. Water evaporation generates mesopores, while partial collapse of unstable macropores reduces spatial heterogeneity. During Stage II (50–100 °C), thermal stress homogenizes surfaces yet generates micropore-driven spatial heterogeneity. During Stage III (100–200 °C), advanced oxidation generates surface defects and irregular micropores, restoring surface roughness. Spatial fractal dimensions stabilize, indicating equilibrium between pore collapse and reconstruction.

**Fig. 11:**
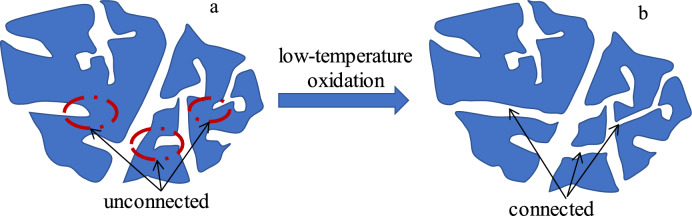
Illustration of the pore structure evolution of oxidized lignite in Lingquan Mine.

In summary, the evolution model of the pore structure of the oxidized lignite from the Lingquan Mine was drawn based on researching the pore volume, specific surface area, micromorphology, and fractal dimension of oxidized lignite from Lingquan Mine, as shown in Fig. 11. The model of the pore structure of raw coal in Lingquan Mine is shown in Fig. 11(a). The model of the pore structure of oxidized lignite in Lingquan Mine is shown in Fig. 11(b). The blocked pores shrink and collapse or expand and rupture, forming channels connecting isolated pores as the oxidation temperature increases.

### Analysis of the microcrystalline structure of oxidized lignite

**Fig. 12:**
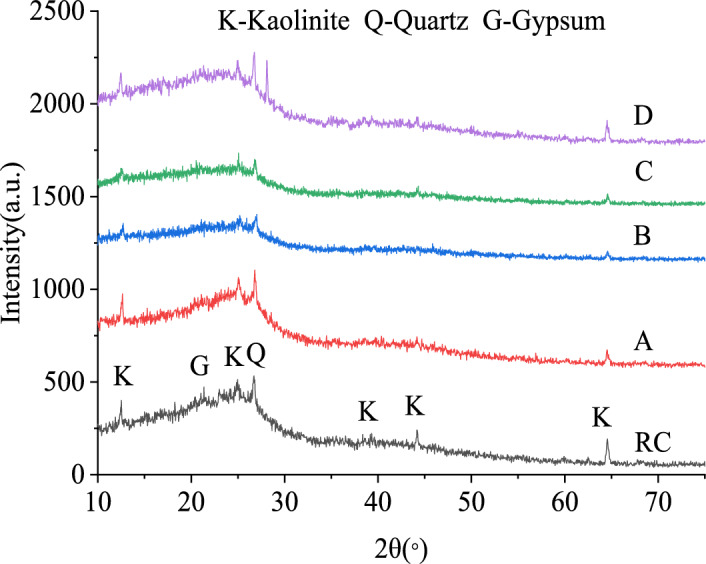
XRD diffraction pattern of oxidized lignite from Lingquan Mine.

The diffraction pattern and mineral composition of oxidized lignite from Lingquan Mine are shown in Fig. 12. Research results show that the X-ray diffraction pattern of oxidized lignite in Lingquan Mine is similar to that of graphite and has the hierarchical structure characteristics of graphite^19,^^63^. The X-ray diffraction pattern of the oxidized lignite in Lingquan Mine shows the central diffraction peak of 002 near 25° and the 100 peak near 45°, as shown in Fig. 12. The 002 peak mainly represents the vertical stacking degree of aromatic rings in the microcrystalline structure of oxidized lignite from Lingquan Mine, and the 100 peak represents the lateral condensation degree of the microcrystalline structure of oxidized lignite from Lingquan Mine. The γ peak on the left side of the 002 peak mainly represents the structure of aliphatic hydrocarbon in oxidized lignite, and the γ peak area is proportional to the content of aliphatic hydrocarbon structure.

**Fig. 13:**
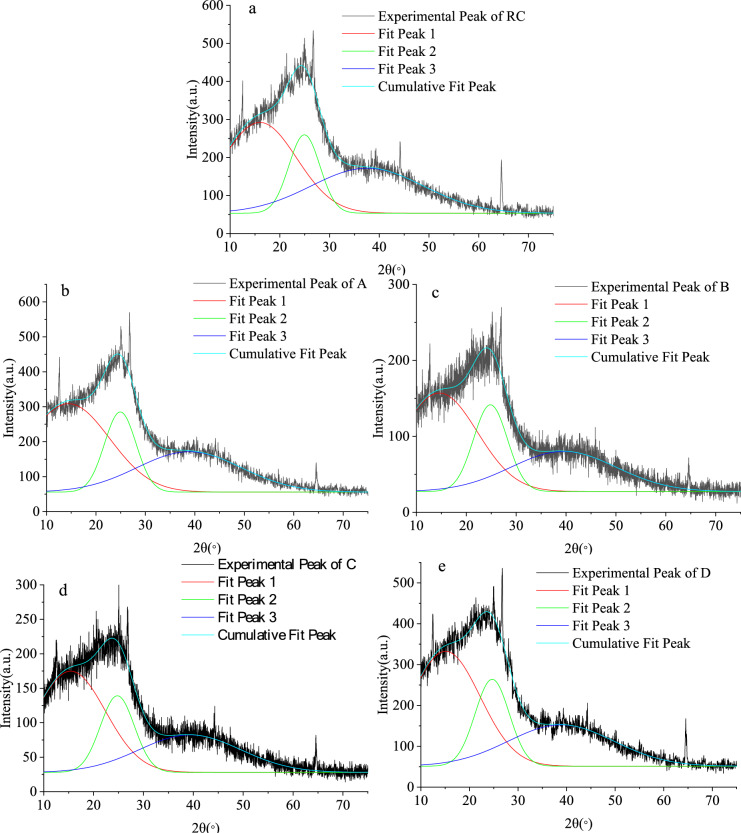
Fitting curve of XRD peak of oxidized lignite from Lingquan Mine.

**Fig. 14:**
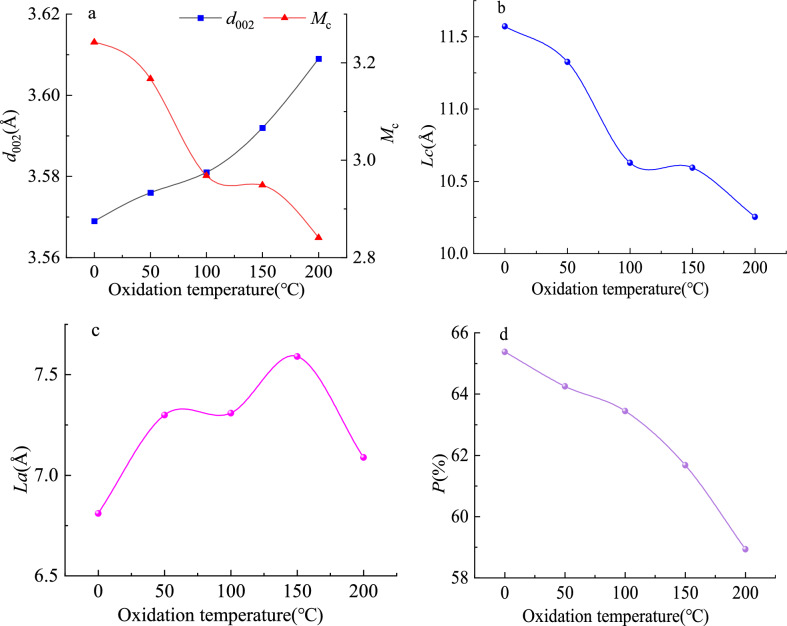
Variational curves of microcrystalline structure parameters of oxidized lignite.

The fitting curve of the XRD peak of oxidized lignite from Lingquan Mine is shown in Fig. 13. The broad peaks of oxidized lignite in the 20 to 50° range were divided into different peaks to obtain γ peak, diffraction angle of 002 peak, full width at half maximum, and other parameters. These parameters were substituted into Eqs. (5)-(10), and the microcrystalline structure parameters *d*_002_, *L*_*c*_, *L*a, *M*_*c*_, and *P* of oxidized lignite in Lingquan Mine were obtained through numerical calculation, as shown in Fig. 14.

The spacing of aromatic layer *d*_002_ of oxidized lignite in Lingquan Mine shows an increasing trend with the increase of oxidation temperature, as shown in Fig. 14 (a). The effect of pre-oxidation temperature on *d*_002_ exhibits a non-monotonic trend, with *d*_002_ showing a significant increase due to the decomposition of oxygen-containing functional groups and the weakening of hydrogen bonds, ultimately leading to the loosening of the microcrystalline structure. The average size of the aromatic layer of oxidized lignite *L*_*a*_ showed a trend of first increasing and then decreasing, as shown in Fig. 14 (c). During pre-oxidation at 0–150 °C, the decomposition of oxygen-containing functional groups and rupture of hydrogen bond networks dominated the structural evolution of coal, which released interlayer constraints and consequently increased *L*_*a*_. In contrast, at 150–200 °C, oxidation-driven disordered radical recombination progressively etched aromatic nuclei, leading to a significant reduction in *L*_*a*_. The number of aromatic layer *M*_*c*_, stacking height of aromatic layer *L*_*c*_, and coalification degree *P* of oxidized lignite show a decreasing trend with the increase of oxidation temperature, as shown in Fig. 14 (a)(b)(d). The pre-oxidation temperature induces the synchronous decrease of *M*_*c*_, *L*_*c*_, and *P* through a chain reaction involving deoxygenation, bond cleavage, and structural collapse. The oxidation reaction progressively disrupts the microcrystalline stacking framework of coal, resulting in a structural transition from an ordered to a disordered state.

The spacing of aromatic layer *d*_002_ of oxidized lignite A, B, C, and D increased by 0.20%, 0.34%, 0.64%, and 1.12%, respectively, compared with raw coal. However, the increase is limited, indicating a difference in the spacing of the aromatic layer of oxidized lignite, but the difference is negligible. The stacking height of aromatic layer *L*_*c*_ of oxidized lignite A, B, C, and D decreased by 2.13%, 8.17%, 8.45%, and 11.39%, respectively, compared with the original coal. The number of aromatic layers *M*_*c*_ of oxidized lignite A, B, C, and D decreased by 2.31%, 8.45%, 9.04%, and 12.37%, respectively. The average aromatic layer size *L*_*a*_ of oxidized lignite A, B, C, and D increased by 7.18%, 7.33%, 11.45%, and 4.10%, respectively. The results of this study show that the number of aromatic rings in the molecular structure of oxidized lignite is lower. The number of aliphatic side chains and oxygen-containing functional groups is more significant, revealing that oxidized lignite has higher reactivity. The coalification degree *P* of oxidized lignite A, B, C, and D decreased by 1.72%, 2.96%, 5.66%, and 9.85%, respectively, indicating that the molecular structure of oxidized lignite is less stable, more susceptible to attack by oxygen molecules, and more prone to spontaneous ignition.

To sum up, the research results show that the higher the oxidation temperature, the lower the stability of the molecular structure of oxidized lignite in Lingquan Mine. Oxygen molecules attack the more easily oxidized lignite, the more likely it is to ignite spontaneously^64,65^.

### Analysis of the functional group and TG of oxidized lignite

**Fig. 15:**
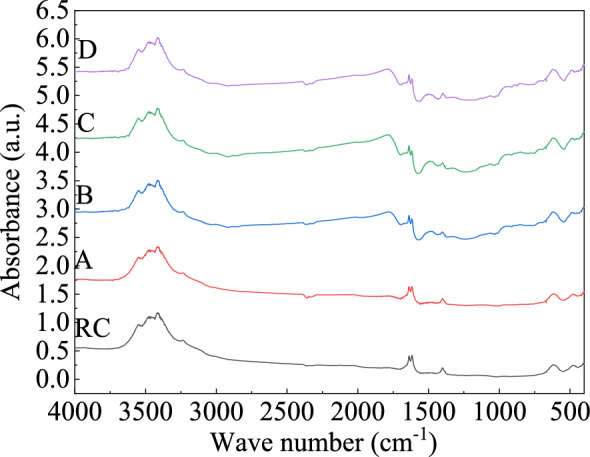
Infrared spectrum curve of oxidized lignite in Lingquan Mine.

**Fig. 16:**
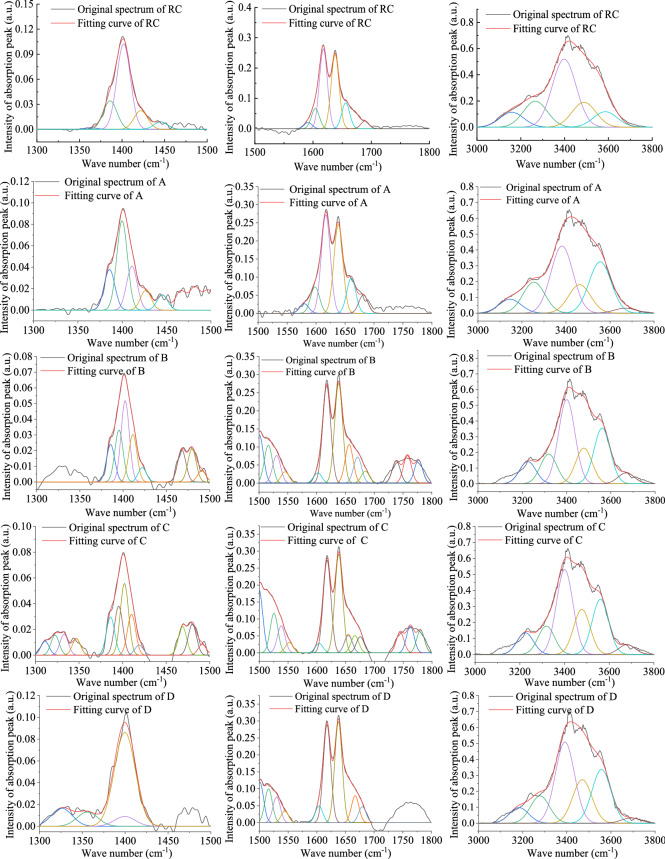
Peak fitting curve of the infrared spectrum of oxidized lignite in Lingquan Mine.

**Fig. 17:**
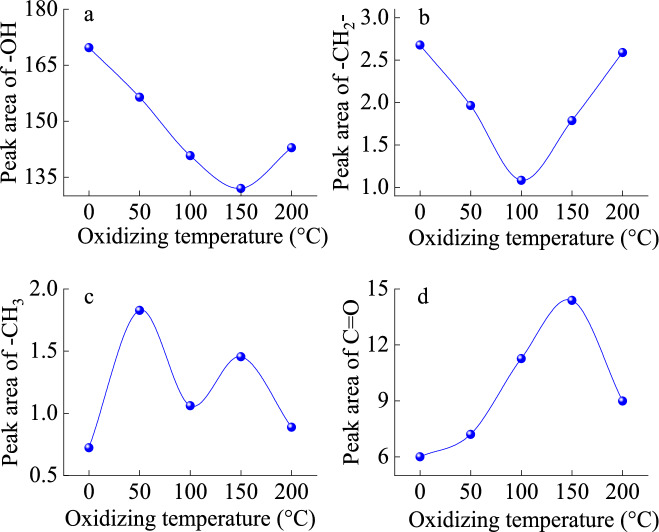
Variational curve of functional group peak area of oxidized lignite in Lingquan Mine.

The infrared spectrum curve of low-temperature oxidized lignite from Lingquan Mine is shown in Fig. 15. The infrared intensity of the absorption peak of oxidized lignite increases compared with that of raw coal. The structures of the infrared spectrum of the oxidized lignite in Lingquan Mine are similar, and the curves of the infrared spectrum are often presented in the form of superposition and a combination of multiple spectral peaks^66^. The spectrum peaks of the oxidized lignite were fitted by combining second-order derivatives, Gaussian, and Lorentz equations with the help of PeakFit software^67,68^. The fitting curves of the infrared spectrum of oxidized lignite from Lingquan Mine were obtained, and the correlation coefficients of the fitting of the infrared spectrum were all greater than 0.99. The characteristic absorption peaks of oxidized lignite in Lingquan Mine are mainly in the range of 1300–1500 cm^−1^, 1500–1800 cm^−1^, and 3000–3800 cm^−1^, as shown in Fig. 16. The variational curves of the functional groups -OH, -CH_2_-, -CH_3,_ and C = O of oxidized lignite in Lingquan mine as the oxidation temperature increases were obtained through numerically calculated^69,70^, as shown in Fig. 17.

#### Variational law of functional group of -OH

The peak area of the -OH functional group of the oxidized lignite from Lingquan Mine first decreases and then increases as the oxidation temperature increases, as shown in Fig. 17(a). The peak areas of functional groups of -OH of oxidized lignite A, B, C, and D decreased by 7.83%, 17.05%, 22.24%, and 15.79%, respectively, compared with raw lignite, as shown in Fig. 17(a). The peak area of the -OH functional group of oxidized lignite C is the smallest. The reason is that the functional group of -OH is highly active and can combine with oxygen to form new functional groups at lower temperatures. The consumption rate of functional groups of -OH is maximum at the oxidation temperature of 150 °C^71,72^.

#### Variational law of functional group of -CH_2_-

The peak area of the -CH_2_- functional group of the oxidized lignite from Lingquan Mine first decreases and then increases as the oxidation temperature increases, as shown in Fig. 17(b). The peak areas of functional groups of -CH_2_- of oxidized lignite A, B, C, and D decreased by 26.67%, 59.60%, 33.34%, and 3.31%, respectively compared with raw lignite, as shown in Fig. 17(b). The peak area of the functional group of -CH_2_- of oxidized lignite B is the smallest. The reason is that the functional group of -CH_2_- is highly active and can easily participate in oxidation reactions. -CH_2_- is consumed in large quantities at 100 °C, and the consumption rate is much greater than the generation rate.

#### Variational law of functional group of -CH_3_

The peak area of the -CH_3_ functional group of the oxidized lignite from Lingquan Mine shows a dynamic nonlinear evolution pattern of first increasing and then decreasing, then increasing and then decreasing as the oxidation temperature increases, as shown in Fig. 17(c). The peak areas of functional groups of -CH_3_ of oxidized lignite A, B, C, and D increased by 152.74%, 46.55%, 101.02%, and 22.68%, respectively, compared with raw lignite, as shown in Fig. 17(c). The peak area of the functional group of -CH_3_ of oxidized lignite D is the smallest. The stretching of the aromatic ring branch chain of oxidized lignite in Lingquan Mine increases the generation and consumption rate of -CH_3_ with increased oxidation temperature^73^. It is worth noting that -CH_3_ is one of the key functional groups that stimulate the spontaneous combustion of oxidized lignite^74–76^.

In summary, the research results show that oxidized lignite releases more CO and CO_2_ than raw coal when it comes into contact with oxygen, making it more susceptible to spontaneous combustion^77^.

#### Variational law of functional group of C = O

The peak area of the C = O functional group of the oxidized lignite from Lingquan Mine first increases and then decreases as the oxidation temperature increases, as shown in Fig. 17(d). The peak areas of functional groups of C = O of oxidized lignite A, B, C, and D increased by 20.09%, 87.86%, 139.78%, and 49.83%, respectively, compared with raw lignite, as shown in Fig. 17(d). The peak area of the functional group of C = O of oxidized lignite B is the largest. The reason is that -CH_2_- and -CH_3_ are oxidized to form groups of C = O, which increases the generation rate of C = O.

In summary, it is more prone to spontaneous combustion and releases more CO and CO_2_ when the secondary oxidation of oxidized lignite in Lingquan Mine occurs.

#### Analysis of TG

**Fig. 18:**
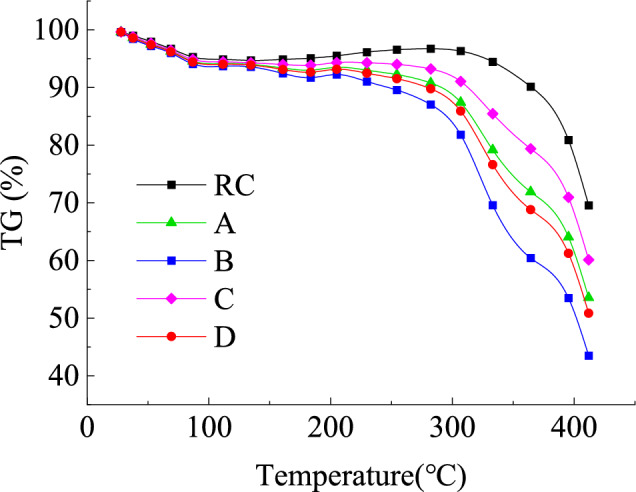
Variational curve of TG of oxidized lignite in Lingquan Mine.

As shown in Fig. 18, non-isothermal thermogravimetric analysis of lignite and its pre-oxidized samples indicates that at a heating rate of 20 °C/min, oxidized coal exhibits better oxidation kinetics during combustion, with a maximum mass loss rate 29.73% higher than that of raw coal. During the low-temperature oxidation stage 0–200 °C, the pre-oxidation treatment has a limited effect on the initial pyrolysis behavior. However, when the temperature enters the main combustion zone 200–400 °C, the decomposition kinetics of oxidized lignite show significant acceleration.

This strengthening effect originates from pre-oxidation-induced coal matrix reorganization. The dissociation of stable macromolecular structures produces highly reactive radical intermediates, which accelerate the formation of peroxy complexes through chain reactions^78^.

As shown in Fig. 17, FTIR quantitative analysis confirms that the preoxidation process triggers significant chemical restructuring: the functional groups—OH and—CH_2_- decrease, while the content of methyl—CH_3_ and carbonyl C = O increases. The synchronous pore evolution analysis showed that the maximum increase in pore volume in the oxidized sample was 3.07 times, and the specific surface area increased by 2.42 times, effectively improving the oxygen diffusion coefficient. This multi-scale coupling mechanism significantly increases the fire risk of oxidized lignite^26^.

## Conclusions

The effect of low-temperature oxidation on the microstructure of lignite in Lingquan Mine was analyzed multi-dimensionally from the pore structure, micromorphology, microcrystalline structure, and functional groups of oxidized lignite through low-temperature nitrogen adsorption, SEM, XRD, and FTIR tests. The conclusions are as follows:

1. The research results show that Mesopores dominate the pore distribution of oxidized lignite in Lingquan Mine, and the low-temperature oxidation process promotes the development of mesopores. It was found that the surface fractal dimension of oxidized lignite first decreased and then increased, which was opposite to the overall trend of mesoporous volume and specific surface area of mesopore. The experimental results demonstrate a synergistic coupling between the spatial fractal dimension, mesopore volume evolution, and surface area reconfiguration in the oxidized lignite, revealing matrix-porosity coevolution mechanisms during the oxidation decomposition process.
2. The research reveals that the aromatic interlayer spacing *d*_002_ of oxidized lignite in Lingquan Mine increases with the increase of oxidation temperature, exceeding raw lignite by 0.20–1.12%. The average size *L*_*a*_ of the aromatic layer initially grows and then declines, showing an overall increase of 4.10%−11.45% compared to the raw lignite. Conversely, the number of aromatic layers *M*_*c*_, stacking height *L*_*c*_, and coalification degree *P* decrease with higher oxidation temperatures, with reductions of 2.31%−12.37%, 2.13%−11.39%, and 1.72%−9.85%, respectively. Analysis of oxidized lignite from Lingquan Mine demonstrated that these structural alterations, characterized by lower coalification degree *P* and looser microcrystalline configuration, significantly diminished molecular structural stability while enhancing spontaneous combustion propensity.
3. FTIR analysis revealed that low-temperature oxidation activates functional group transformation in Lingquan Mine lignite, elevating its reactivity. Quantitative deconvolution showed temperature-dependent nonlinear evolution of key moieties: Hydroxyl -OH and methylene -CH_2_- groups decreased by 7.83–22.24% and 3.31–59.60%, respectively. Methyl -CH_3_ and carbonyl C = O groups increased substantially by 22.68–152.74% and 20.09–139.78%. This selective enrichment of electron-donating -CH_3_ and oxygen-containing C = O groups establishes radical reaction pathways, synergistically enhancing chain reaction dynamics and spontaneous combustion propensity.

## Data Availability

Te datasets used and/or analysed during the current study available from the corresponding author on reasonable request.
